# The dynamics of family planning and abortion services during COVID-19: perspectives from healthcare providers and clients in Burkina Faso

**DOI:** 10.1186/s12978-026-02340-x

**Published:** 2026-07-21

**Authors:** Eunice Nahyuha Chomi, Marie Gisèle Donessouné, Armel Sogo, Armando Humberto Seuc, Gabriela Garcia Camacho, Ali Moazzam, Seni Kouanda, the WHO HRP Social Science Research Team, Luis Bahamondes, Jose Guilherme Cecatti, Vilma Zotareli, Rachel E. Soeiro, Karayna G. Fernandes, Mariana B. Rogerio, Samira M. Haddad, Silvana F. Bento, Karla S. Padua, Aline Munezero, Charles M. Charles, Montas Laporte, Eunice Chomi, Flore Marie Gisèle Donessouné, Kun Tang, Yueping Guo, Hanxiyue Zhang, Yifan Zhu, Ge Yang, Chunxiao Peng, Xizhuo Xie, Hao Wang, Deda Ogum Alangea, Kwasi Torpey, Emefa Judith Modey, Adom Manu, Ernest T. Maya, Rozina Karmaliani, Laila Ladak, Arusa Lakhani, Marina Baig, Yasmin Parpio, Salima Somani, Pisake Lumbiganon, Jen Sothornwit, Nampet Jampathong, Somporn Rungreangkulkij, Marleen Temmerman, Ferdinand Okwaro, Abdu Mohiddin, Massimo Mirandola, Maddalena Cordioli, Alessia Savoldi, Simone Garzon, Stefano Uccella, Nigel Sherri, Alexandra Sawyer, Catherine Aicken, Jörg W. Huber, Jaime Vera, Deborah Williams, Moazzam Ali, Caron Kim, Vanessa Brizuela, Hamsadvani Kuganantham, Grace Kapustianyk, Igor Toskin, Soe Soe Thwin, Armando Seuc

**Affiliations:** 1 Reproductive Health Unit, Institute for Research in Health Sciences, Ouagadougou, Burkina Faso; 2 Department of Sexual and Reproductive Health, World Health Organization, Geneve, Switzerland

**Keywords:** Family planning services, Abortion services, Complications of abortion, COVID-19, Service disruption, Service delivery

## Abstract

**Background:**

The novel SARS-CoV-2 (COVID-19) led to an unprecedented increase in demand on health systems to care for people infected, necessitating the allocation of significant resources, especially medical resources, towards the response. This, compounded by the restrictions on movement led to disruptions in the provision of essential services, including sexual and reproductive health (SRH) services. This study assessed the effects of the COVID-19 pandemic on demand, utilisation and delivery of family planning and abortion services in Burkina Faso.

**Methods:**

The study used a mixed method, cross-sectional panel survey to collect data at two time points in eight health facilities in Ouagadogou and Bobo Dioulasso. Data was collected using in-depth interviews (IDI) and focus group discussion (FGDs) with women, their male partners, and healthcare providers and health facility availability and readiness assessments.

**Results:**

Demand for family planning services declined during the lockdown period due to movement restrictions, fear of infection and of being quarantined if found to be infected. Despite no direct demand for abortion services, healthcare providers reported increased unintended pregnancies and demand for treatment of complications of abortion, suggesting recourse to unsafe abortions. At least 30% of facilities experienced partial disruption in delivery of services, including staff shortages, supply chain challenges and reduced outpatient volumes. Health facility readiness scores remained moderate, implying existing constraints worsened by the pandemic.

**Conclusions:**

COVID-19 response and containment measures indirectly affected demand, delivery and utilisation of family planning and abortion services in Burkina Faso, by increasing the existing barriers for women and girls. Strengthening health system resilience, community-based service delivery and communication strategies is essential to ensure uninterrupted access during health emergencies.

**Supplementary Information:**

The online version contains supplementary material available at 10.1186/s12978-026-02340-x.

## Abstract (plain language summary)

Our study assessed the effects of the COVID-19 pandemic on the demand, utilisation and delivery of family planning and abortion services in Burkina Faso. The study was carried out at the health facility and involved women seeking family planning or abortion services, their partners and healthcare providers. Data was collected and analysed using qualitative and quantitative methods. Demand for family planning decreased mainly due to fear of infection and fear of being tested and subsequently quarantined if found to be infected. While there was no direct demand for abortion services, there was a surge in demand for treatment of complications of abortion and unintended pregnancies. Containment measures introduced during the period of high transmission disrupted service delivery, which in turn affected demand and utilisation of family planning services. This study has highlighted the negative consequences of COVID-19 containment measures on delivery, demand and utilisation of family planning and abortion services by further reducing the capacity to deliver services and increasing the existing barriers for women and girls.

**Supplementary Information:**

The online version contains supplementary material available at 10.1186/s12978-026-02340-x.

## Background

The SARS-CoV-2 (COVID-19) pandemic led to unprecedented demands on health systems, necessitating the reorganisation of resources to maintain the continuity of essential health services while managing COVID-19 cases and preventing both direct and indirect mortality [1]. This put tremendous pressure on health systems, that served to further burden healthcare systems already grappling with scarce resources and worsened the availability and inequity of access to services. Public health measures instituted in response to the pandemic, such as lockdowns, and shutdown of non-essential services severely disrupted daily lives and livelihoods and reduced accessibility of essential health services [2, 3]. The potential consequences of such disruptions on health outcomes, including sexual and reproductive health (SRH) of women and girls were expected to be devasting. For instance, lessons from the Ebola and Zika virus outbreaks have highlighted severe disruptions in SRH services that expose women and girls to preventable health risks [4, 5]. Furthermore, the UNFPA projected that the pandemic would result in a reduction in contraceptive use and an increase in unintended pregnancies, particularly in low- and middle-income countries [6]. More recently, studies have documented the negative effect of COVID-19 on disruptions to SRH service delivery and access, including the gendered impacts of the pandemic that worsened the existing unequal gender relations and further weakened the health system’s capacity to deliver services [7–15].

Burkina Faso experienced several peaks in COVID-19 transmission between 2020 and 2021, with the most peaks occurring in 2020 [16]. Containment measures included closure of all borders and restricted domestic mobility, physical and social distancing measures, and isolation with a dusk to dawn curfew [17]. The country experienced a lower burden and shorter period of disruptions to service delivery and everyday life. However, considering the pre-existing health system weakness, the costs of both direct and indirect effects of the pandemic may be greater, with potentially devasting consequences for public health.

Family planning services are provided by level 1 and 2 primary health facilities, while abortion services are provided by level 2 facilities. Referral cases are treated at level 3 regional facilities and specialised services are provided at the University teaching hospitals. Under the *Gratuité*, family planning services are provided free of charge [18]. Understanding the level of service disruption, and how this affected demand and service delivery in the Burkina Faso context is important to ensure continuity of services in future health emergencies. Several studies have assessed the effects of COVID-19 on disruption of SRH services in Burkina Faso, focusing mostly on the magnitude, dynamics and determinants [19–22] but have not captured the healthcare provider and women’s perspectives on delivery, demand and utilisation of the services.

The aim of our study was to assess the level of disruption of family planning and abortion services and to explore the perspectives of women and healthcare providers as they experienced the service disruptions and effects of COVID-19. Our study was carried out as part of a wider study on the effects of COVID-19 on the availability and readiness of health systems in Brazil, Burkina Faso, Kenya, Ghana, Italy, Pakistan, Thailand and the United Kingdom.

## Methods

### Study design

This was a cross-sectional panel survey with two data collection points, 6 months apart, using mixed methods to assess the availability, readiness and utilisation of family planning and abortion services during and following the COVID-19 pandemic. The qualitative component used semi-structured in-depth interviews (IDIs) and focus group discussions (FGDs) to examine clients’ and healthcare providers’ perceptions of risk, access barriers, and utilisation of family planning during and after the COVID-19 pandemic. The quantitative component used a health facility assessment to assess availability, readiness and utilisation of family planning and abortion services during and after the COVID-19 pandemic.

At baseline, the first round of data collection was designed to represent the period of high transmission (during pandemic), while at endline, the second round represented the period of recovery (post-pandemic), where transmission levels had stabilised. The two rounds of data collection were designed to allow for documentation of family planning and abortion services during the pandemic and assessment of changes, adaptations and recovery over time without following the same individual participants longitudinally. For both components, the first round of data collection was conducted from February to May 2022, and the second round was collected from December 2022 to March 2023.

The timing of data collection was guided by the World Health Organisation (WHO) situation-level assessment of COVID-19 transmission, which categorises epidemic status from level 0 (no transmission) to level 4 (widespread epidemic) [23].

### Study setting

The study was carried out in the cities of Bobo Dialousso and Ouagadougou, which had the highest transmission rates. This was a facility-based study, carried out in 8 health facilities: 3 University teaching hospitals (*CHUs*) and 5 district health facilities (*CMAs*). We selected 2 of the *CHUs* and 3 of the CMAs from Ouagadogou since it experienced higher transmission rates and has more health facilities than Bobo Dialousso. The facilities were selected based on their ability to provide family planning and abortion services and because they serve a heterogeneous mix of patients from diverse socio-economic backgrounds. All facilities were urban based because the transmission in Burkina Faso was concentrated in urban areas, with limited spill over to rural facilities.

### Study population

Study participants were women seeking family planning and abortion services from the selected health facilities, their accompanying partners and the healthcare providers working in those facilities. Selection of participants was performed at both rounds of data collection, following the same process and with a new sample at each round.

### Qualitative methods

#### Sampling, selection and recruitment

The women were purposively selected based on 1) age (18–49 years) and 2) whether they were seeking SRH services, while their partners were selected based on age (18–49 years). Potential participants were screened by study gatekeepers, then introduced to the research team if they agreed to participate in the study. The target sample size was 18 clients (women and accompanying partners) per facility or until data saturation was achieved. A total of 16 healthcare providers (2 per facility) were purposively selected based on whether they were currently delivering SRH services and had been working at the clinic for at least 6 months before the pandemic started.

#### Data collection

Data was collected by eight researchers with prior experience in conducting qualitative research on sensitive SRH topics. The researchers received training specific for this study, with a special emphasis on ethical conduct, rapport building and non-judgemental interviewing. To reduce power imbalances and enhance participant comfort, interviewers were matched with participants based on their gender and fluency of the local language. The data collection team included social scientists and public health researchers with experience in sexual and reproductive health and health systems research and did not have prior relationships with study participants.

The study tools were adapted from the generic tools developed for the wider study, pretested and included interview guides specific to each participant group: 1 FGD guide for women, 1 interview guide each for men and women, and 1 interview guide for healthcare providers. The tools for women explored their knowledge about COVID-19, risk perceptions and concerns about effects on family planning and abortion, care-seeking behaviours, experience, and barriers in accessing the services. For men, we explored their knowledge of COVID-19, risk perceptions and concerns about effects on SRH, their influence on access to family planning and abortion services of their partners and what role they played when their partners required the services. In-depth interviews with healthcare providers focused on their perceptions of demand for, and delivery of family planning and abortion services during COVID-19.

All study participants underwent a detailed informed consent prior to participation and audio recording. Interviews were carried out in a private, secure room at the health facility to ensure confidentiality. All interviews were audio-recorded, lasted 40–60 min and were conducted in two local languages (Jula or Mòoré) for clients and in French for healthcare providers. Data collection continued until saturation was reached.

No repeat interviews were conducted as the study was designed as a cross-sectional panel with different samples at each data collection round. Refusals to participate were minimal and were primarily due to time constraints. Field notes were systematically collected during interviews.

#### Data management and analysis

All interviews were digitally recorded, transcribed verbatim and de-identified using unique participant codes. Audio files and transcripts were encrypted and stored in a secure virtual repository at the Institut de Recherche en Sciences de la Santé (IRSS) and shared with WHO. Only the research team were authorised to access to the recordings and transcripts.

Jula and Mòoré transcripts were translated into French and back translated to ensure accuracy by research team members fluent in either Jula or Mòoré and French. All transcripts were imported into NVivo and data from women, partners and healthcare providers were merged into a single analytical dataset. Data were analysed using a hybrid deductive-inductive thematic analysis approach. An initial deductive codebook was developed by coders with expertise in social sciences and public health based on study objectives and the conceptual focus on women’s demand for and experiences of accessing and utilising family planning and abortion services, and healthcare providers’ perceptions of demand and their experiences in service delivery. The inductive approach allowed for emergence of new codes from the data, to capture unanticipated issues and nuances not predefined in the analytical framework.

Two members of the team separately coded an initial subset of transcripts and then jointly refined the codebook after discussion of discrepancies. The coding scheme was iteratively refined through constant comparison across transcripts. The revised codebook was then applied to the full dataset, and codes were organised into themes and subthemes reflecting demand for services, access barriers, service adaptations and care seeking experiences. Transcripts were coded using Nvivo 12. The analysis focused on the demand for and experiences of the women in accessing and utilising family planning and abortion services; and the healthcare provider’s perceptions on the demand for and their experiences in delivering the services. Analysis was stopped when no additional codes or themes emerged. NVivo comparison tools were used to examine similarities and differences between baseline and endline data. The transcripts and findings were not reviewed by participants.

### Quantitative methods

#### Sampling, selection and recruitment

In each health facility, one healthcare provider who was most knowledgeable about SRH services was selected in each facility to assist in completing the health facility assessment questionnaire. Participants in the quantitative component were distinct from those in the qualitative component.

#### Data collection

The health facility assessment questionnaire was adapted from the WHO Service availability and Readiness Assessment (SARA), WHO Rapid Hospital Readiness checklist and the WHO safe abortions assessment [24–26]. The questionnaire assessed availability of services and tracer items for family planning and abortion, the level of service disruption in the health facilities, reasons for the disruption and mitigation measures. Service disruption was categorised as no disruption, partial disruption, or complete disruption.

All study participants provided informed consent prior to data collection. Trained research team members conducted the facility assessment using the tools. Data quality was ensured through daily checks for completeness and logic, weekly supervisory visits and double data entry into OpenClinica by data clerks.

#### Data analysis

Data were analysed centrally by the study statistician at the World Health Organisation for all country study sites using descriptive statistics (frequencies and percentages). Health facility readiness was analysed separately on site, where a service was considered available if it was offered by the facility or received by the clients seeking the service. A tracer item was considered available if it was present at the facility at the time of data collection. Service specific availability was calculated as the total number of facilities offering family planning or abortion services. Readiness was calculated for the facilities that offered family planning and abortion services, as the sum of the availabilities of all the items, divided by the total number of items, then multiplied by 100.

### Ethical considerations

Ethical approval was obtained from Comité d’éthique de Recherché en Santé du Burkina Faso (CERS) in Burkina Faso (No. 2021–01–008), RP2 and WHO Ethics and Research Committee (ID-CERC.0103). Permission to conduct the study in the health facilities was obtained from the health district offices and health facilities. Written consent for participation and recording (for FGDs and interviews) was sought from each participant after undergoing a detailed informed consent process prior to data collection, where they were informed about the purpose of the study, the institutional affiliations of the research team and the voluntary nature of participation. Adherence to all ethical considerations, including voluntary participation, with adherence of confidentiality, anonymity and the right to withdraw without consequences was ensured.

## Results

For the qualitative results, emerging themes in baseline and endline data were very similar, with no new information emerging from the endline data. Baseline and endline data are summarised and presented together.

### Characteristics of the sample

As presented in Table 1, the total sample for both baseline and endline data collection points comprised 86 clients and 16 healthcare providers. Most clients (80%) were aged between 20 and 39 years and married. A third (31%) were educated up to secondary school and 47% were self-employed. Healthcare providers were mostly male (69%) midwives or nurses (88%).

**Table 1:** Characteristics of the qualitative sample

Clients (women and partners, *n* = 86)	N	%
Location		
Ouagadougou	64	74.4
Bobo Dioulasso	22	25.6
Sex		
Male	21	24.4
Female	65	75.6
Age (years)		
≤ 19	6	6.9
20–29	35	40.7
30–39	34	39.5
40 +	11	12.8
Marital Status		
Single	16	20
Married	64	80
Educational Level		
No education	24	32
Primary or less	15	20
Secondary	27	36
University	9	12
Employment Status		
Unemployed	9	11.5
Salaried worker Office worker	26	33.3
Self-employed	37	47.4
Student	6	7.7

### Demand for family planning and abortion services

The demand for family planning services decreased particularly during the lockdown period, with some women continuing to view family planning as a priority need, while others relegated it to less of a priority compared to other needs, like ante-natal care.

"For family planning methods, as I do not use them, I do not think that it is an obligation. We can organise ourselves to avoid a pregnancy while waiting for a solution to be found, but if you are sitting with a pregnancy, it is obligatory to carry out checks and [weight measurements]”. Pregnant woman1, CMA Kossodo.

The number of family planning clients declined because of fear of being infected with COVID-19 or tested and quarantined if found positive. This was especially the case during high transmission where screening stations were set up at the entrance of all health facilities.

"It must be said that during the pandemic, there were no crowds because people had a phobia of the hospital. Some people who had a cough or a cold refused to come to the hospital because they didn't want to be told that they had COVID.” Provider1 Diafra.

The women did not express the need for abortion services during COVID-19, nor did the pandemic change their attitudes towards abortion. The prevailing general sentiment was that abortion was morally wrong, with all women saying they would never desire or seek an abortion despite some getting pregnant unexpectedly while they still had young babies. Interestingly, healthcare providers reported that while there was no direct demand for abortions services, there was a surge in the demand for treatment of complications of abortion.

"During the period of COVID, hiéé [expressive interjection in local language to denote alarm] those who decided to abort were too much, were so much". Provider1 Tengandogo

"In the statistics I saw it [cases of treatment for complications of abortion] was high". Provider1 Bogodogo

Furthermore, the number of unintended or undesired pregnancies also increased, with healthcare providers also reporting a rise in the number of women who desired to terminate their pregnancies but did not demand an abortion. Though not verbally communicated, the women showed the providers that they had no choice but to see their pregnancy through to delivery. A nurse said:

"During the COVID period, we cannot talk about women who intend to have an abortion; there was an upsurge of non-desired pregnancies and they wished to interrupt the pregnancy”*.* Provider2 Tengandogo.

Lockdown measures instituted during the period of high transmission resulted in men spending longer periods in the home, with nothing to do, resulting in increased pressure for sex, according to one healthcare provider:

"It was rather the pregnancies that occurred during the COVID period, and the women complained that their men were at home all the time, so it wasn't ...it wasn't easy.” Provider1 Tengandogo

### Utilisation of family planning and abortion services

The experiences of women seeking and accessing family planning services varied. Even before COVID-19, many of the women only visited health facilities for initiation of a family planning method, then for continuation preferred the services from non-profit organisations that promote family planning, where they didn’t have to spend long hours waiting. For these women there didn’t seem to be any challenges in accessing family planning services.

"No, I haven't had any difficulties accessing the youth watch centre [non-profit organisation promoting family planning], it's even there that I go for the family planning product, I haven't had any difficulties”. Woman1, Tengandogo

For women that relied on health facilities, the lockdown period was challenging because of reported disruptions in service delivery. Measures to reduce transmission such as reducing the number of consultations per day, reducing the number of patients entering the facility at a given time, mask mandates, staff reassignment and staff shortages, and combining clinics and reducing hours of operation/number of clients seen per day to manage staff shortages made access to services difficult.

"It's the number [of patients seen per day] they have decreased. When I leave, it's early now. [You must leave home early, otherwise you won’t receive services]. [To access services, you have to go early; you have to go at 4 am]". Woman1, Bogodogo

Movement restrictions and the imposed curfew, which reduced travel time also made visiting facilities difficult. For some women, movement restrictions kept their spouses at home most of the time, making it difficult to seek family planning services, especially for those who used contraceptives without the knowledge of their spouses.

"Now when they introduced the curfew, [you should be at the clinic by 5.40 am, since they only take 20 people] per day. If you arrive at 6am, you can't [receive services]. You must come back the next day. At 20 people they stop. And from 5am, they [healthcare providers] often come at 8am, often even after 8am”. Woman2, Bogodogo

Shortages of contraceptives were also reported, where the women were given a prescription to purchase them at a higher cost from private pharmacies, posing a financial barrier to contraceptive access.

"The high cost of contraceptives at the pharmacy is an obstacle to being able to afford pills at a lower cost, so the break in supply meant that I got them at the pharmacy, but at a higher price”. Woman2, Tengandogo

Since the women interviewed reported never to have desired or sought an abortion, we could not report on the experiences of access and utilisation of the services.

### Delivery of family planning and abortion services

Family planning services continued to be delivered in all health facilities except Tengandogo *CHU*, which was the first to be designated as a COVID-19 treatment centre and where the services were suspended. Facilities that continued providing family planning services experienced partial disruptions due to staff shortages, risk perceptions of patients and measures introduced to reduce the risk of transmission between patients. For instance, the number of patients seen per day was reduced to allow for proper screening prior to entering the health facility and physical distancing in the waiting areas.

"There were disruptions, it's like I told you, in time we limited the number of patients so that they would not be close together to avoid contamination, so we limited the number of patients per day”. The team is still working, the team is still receiving women, the only thing I can say is that attendance at the beginning decreased because people preferred to stay at home. The women themselves did not even come anymore". Provider3 Yalgado

In Burkina Faso, induced abortion is permitted only in cases of rape, incest or when the life of the woman is endangered by the pregnancy. Therefore, safe abortion services continued to be available, but only under the grounds permitted by law. The healthcare workers reported an increase in the demand for treatment of complications of abortion, which they continued to provide without judgement, saying:

"But as we are not a service that offers this kind of care, it is difficult to answer this question. There are people who come to us for care after their abortion. And we are not gendarmes or policemen. We are there to save; we are there to treat. We are not here to judge”. Provider2 Bogodogo

"We deal with the consequences. People go and do things [receive abortions] elsewhere in conditions that are not safe, and we manage the consequences" Provider2 Yalgado

### Health facility readiness and disruption in family planning and abortion service delivery

#### Service availability and Health facility readiness

**Fig. 1:**
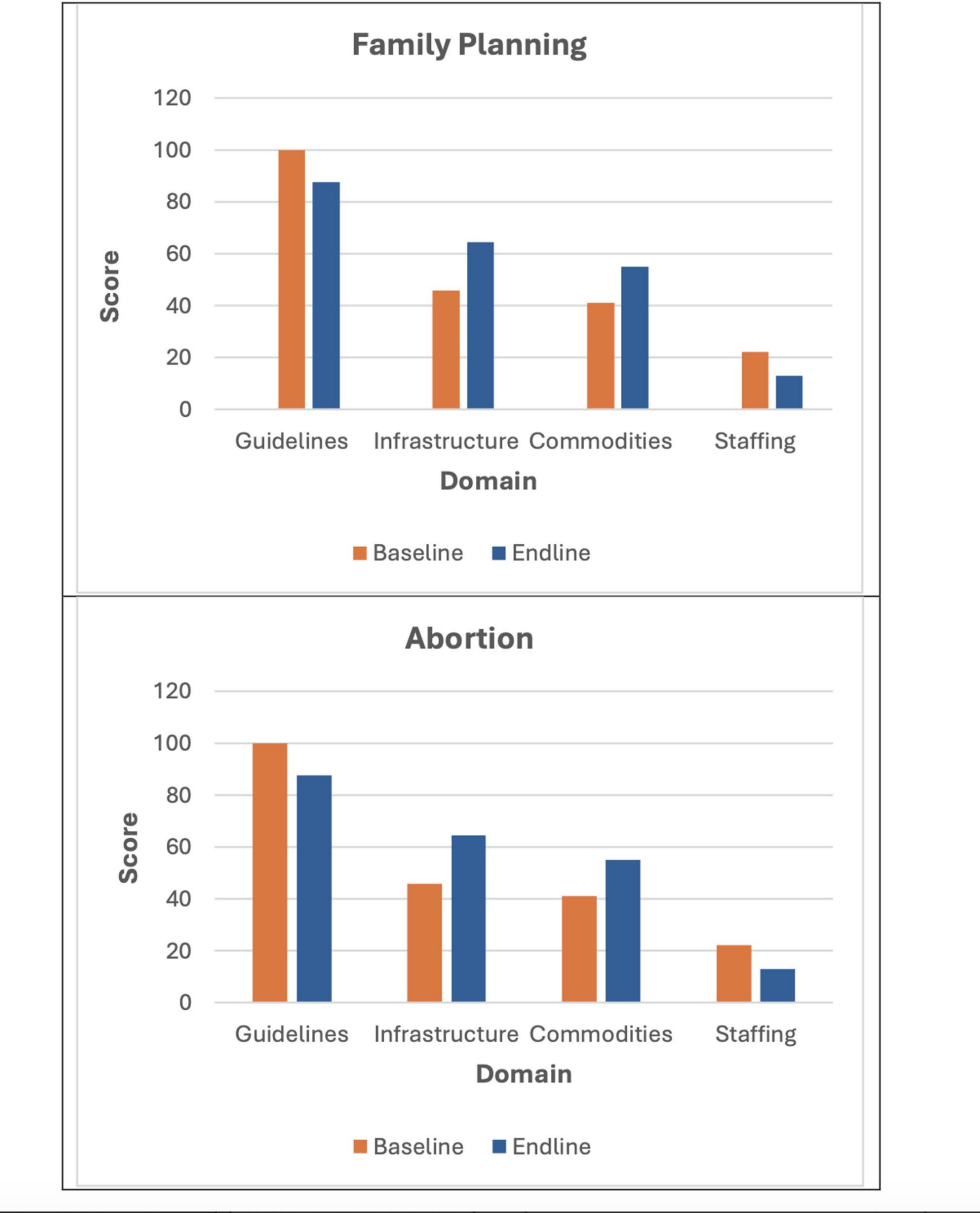
Family planning and abortion domain scores, baseline and endline

All facilities offered family planning services and stock contraceptives. There were no referrals to higher level or other facilities. The highest domain scores were achieved for contraceptive guidelines, with a score of 100 at baseline and 87.5 at endline, while staffing had the lowest score, with 69 at baseline and 50 at endline (Fig. 1, details in Additional file1).

While all facilities stock contraceptive commodities, the average stock-out reported in the past 6 months was 75.9% at baseline and 89.8% at endline (details in Additional file 1). Combined oestrogen progesterone oral contraceptives, progestin only injectable contraceptives, male and female condoms and copper IUD were reported out of stock in all facilities. The readiness score for family planning was similar at baseline (69.4) and endline (69.3).

All facilities offered treatment for complications of abortion. Only one facility reported referrals to higher level or other facilities at baseline, while there were no referrals at endline. The highest domain score was recorded for guidelines, with a score of 100 at baseline and 85.7 at endline, while staffing recorded the lowest: 22 at baseline and 13 at endline (Fig. 1, details in Additional file 2).

**Fig. 2:**
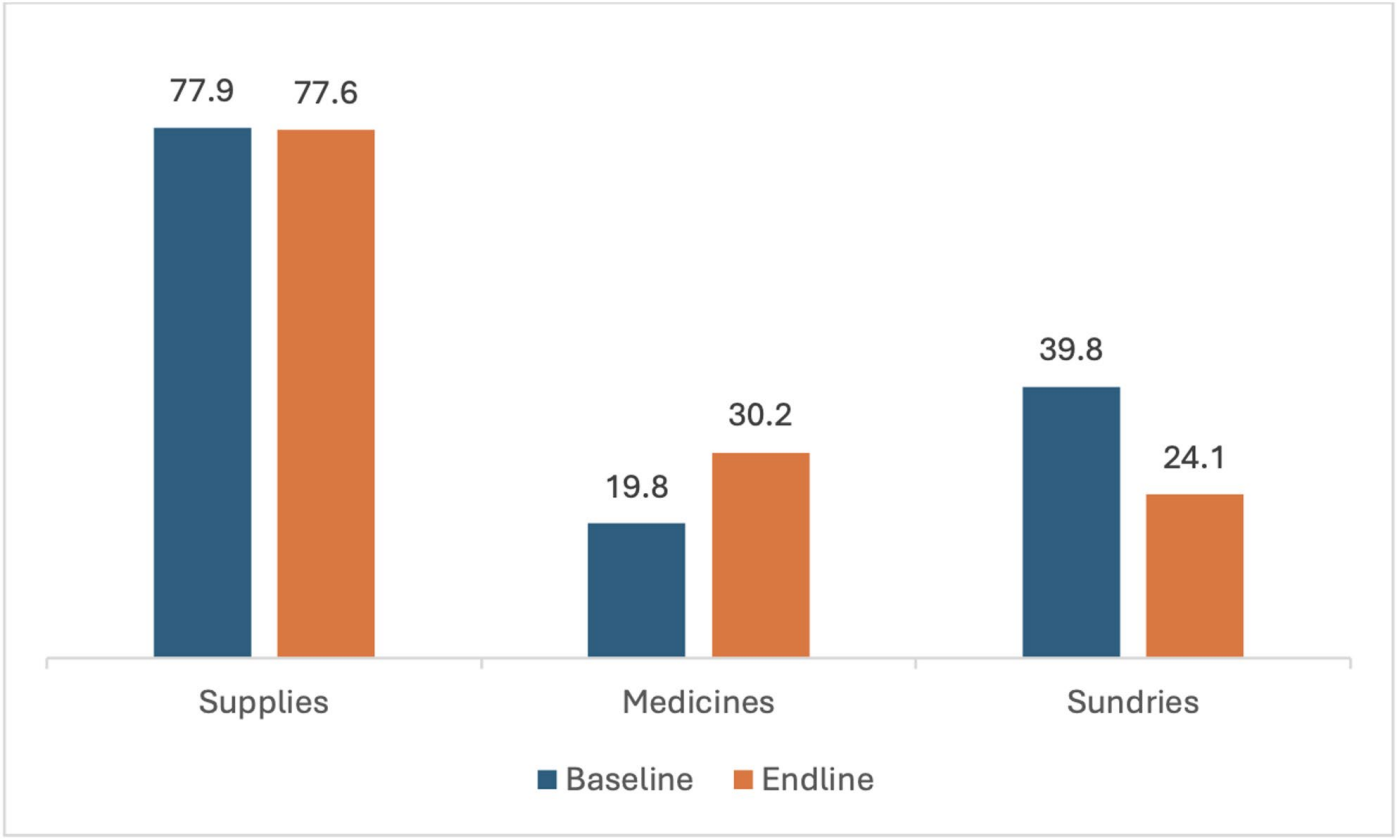
Stockout of abortion commodities, baseline and endline

Availability of essential abortion commodities (abortion supplies, medicines and sundries) was moderate, with 62.5% of facilities having them in stock on the day of the assessment. A high percentage of facilities had stock-outs of abortion commodities with supplies in the last 6 months (Fig. 2, details in Additional file 2). The readiness score was similar at baseline (55) and endline (55.7).

### Service disruption

Healthcare providers reported that contraceptive services and treatment for complications of abortion continued to be delivered in all health facilities except Tengandogo *CHU*, where the services were suspended. Facilities that continued providing services experienced partial disruptions due to staff shortages, risk perceptions of patients and other measures to reduce the risk of transmission between patients.

"People thought that it was in the hospital where one could get infected, especially at the beginning we had put a tarpaulin [screening tent] at the entrance of the hospital where we tested people, people thought that the more you go to the hospital, the more you will get it". Provider1 SS.

During the period of high transmission, staffing of the COVID-19 treatment centres was prioritized to ensure adequate staffing to care for patients. Staff reallocation to the centres was done not only for initial staffing, but also to replace staff that were infected and had to be isolated, resulting in fewer staff and increased workload.

"What we can say is that it lengthens the working time, for example, if there are two of you doing a job and you end up doing it alone, so it will take a lot of time”. Provider2 Diafra

**Fig. 3:**
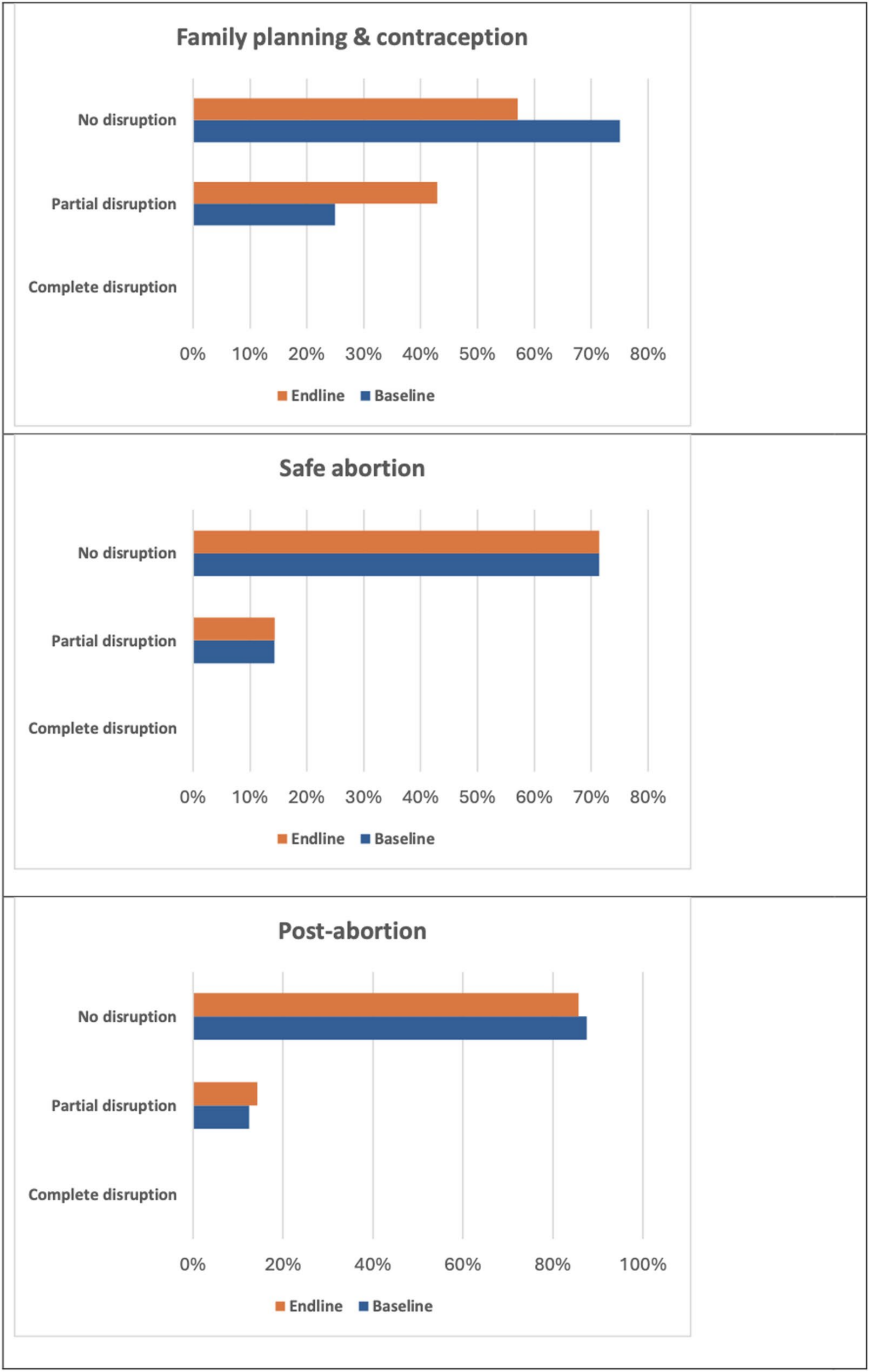
Percentage of facilities with disruptions in family planning and abortion service delivery, baseline and endline

The health facility assessment showed that over 70% of the facilities reported no disruption, while less than 30% reported partial disruption. The results were generally similar for baseline and endline, except for family planning, where more than 40% of the facilities reported partial disruption at baseline and 57% at endline (Fig. 3).

**Fig. 4:**
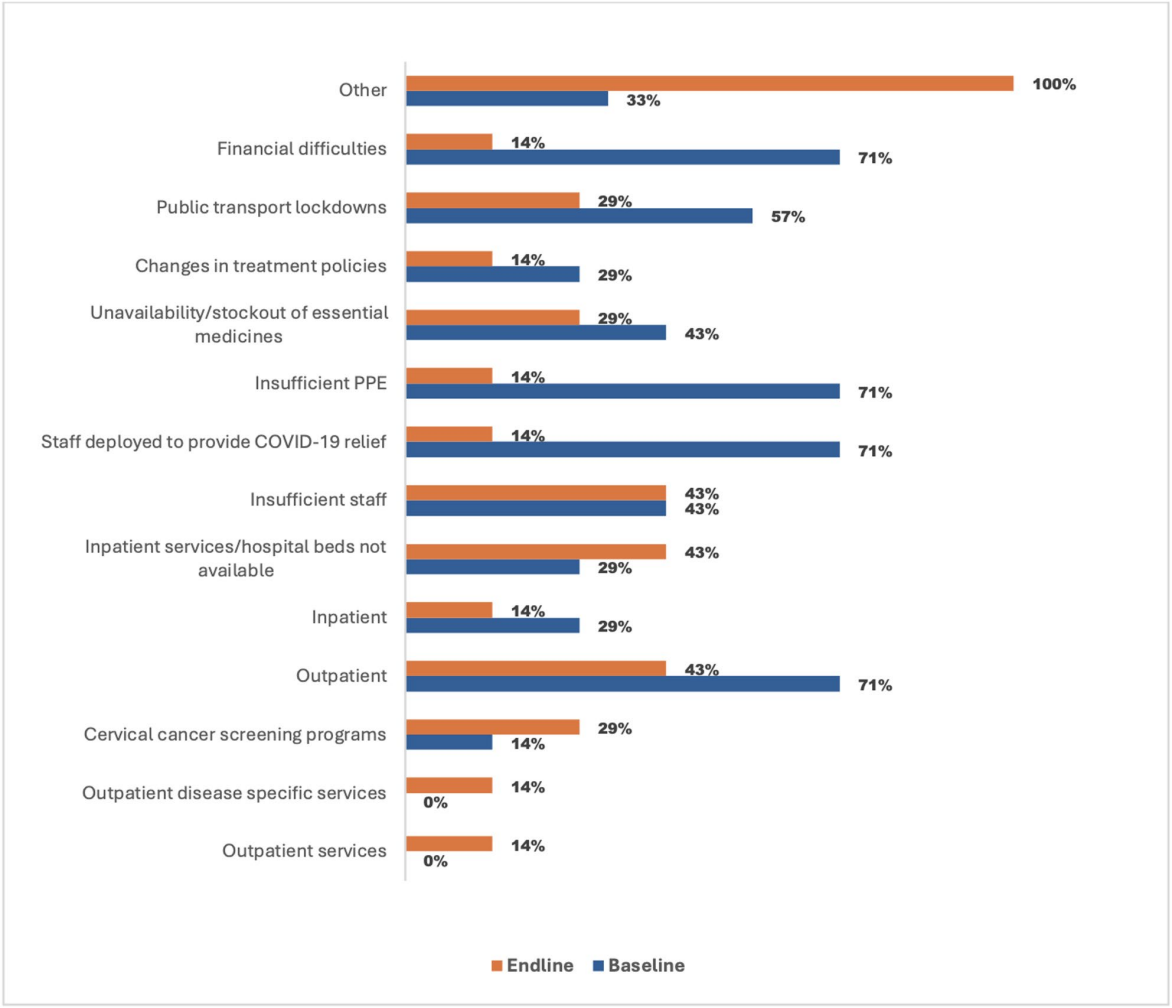
Reasons for disruptions in service delivery, baseline and endline

Aligned with qualitative findings, the main reasons for partial disruption in services at baseline were decreased outpatient volumes, staff deployed to provide COVID-19 relief, insufficient PPE available for health care providers and financial difficulties, reported by 71.4% of the facilities. At endline, the main reason, cited as ‘other’ by all facilities was not specifically related to COVID-19 (Fig. 4).

**Fig. 5:**
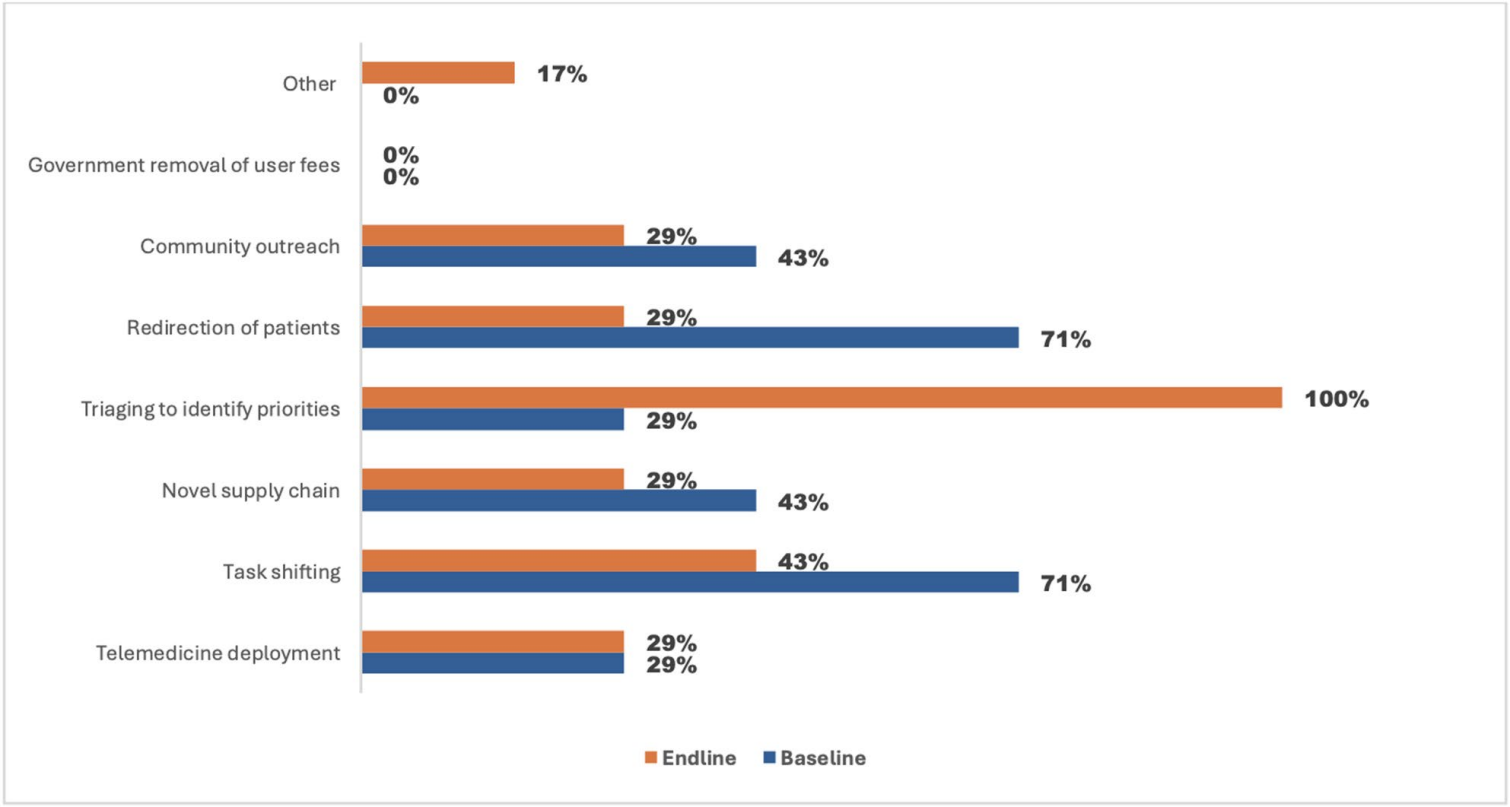
Approaches to overcome service disruption, baseline and endline

Baseline results show mitigation measures similar to those reported in the qualitative findings, mainly task shifting/role delegation and redirection of patients to alternate health care facilities, reported by 71.4% of the facilities. At endline, triaging to identify priorities was the main approach, reported by all facilities (Fig. 5).

## Discussion

Demand for family planning services declined, particularly during the lockdown period. The main reason was the fear of infection, which aligns with previous research [27–30]. Experience from infectious disease outbreaks like Ebola [31, 32] and more recently COVID-19 pandemic [33–36] indicates that the fear of infection can negatively affect health seeking behaviour. Such fears may not be unfounded given the high risk of exposure and infection at health facilities [31, 37], and suggests a lack of confidence in the health system capacity to protect patients while providing essential services during disease outbreaks [11, 35]. The fear of being tested and quarantined if found infected was also reported [17, 38]. Taken together, these findings imply a suppressed demand for family planning services and highlight the importance of strengthening the preparedness measures during future outbreaks. Information, education and communication (IEC) campaigns with messaging tailored to allay such fears and provide information on accessing services will promote sustained demand for services.

Our findings on the demand for abortion services are not very straightforward. On the one hand, the attitude towards abortion, based mainly on religious and moral values, was not affected by COVID-19, nor was the demand for abortion services. On the other hand, healthcare providers reported a surge in demand for treatment for complications of abortion, coupled with an increase in unintended pregnancies and an unspoken desire for termination of pregnancy or decision to keep the pregnancy out of compulsion. This implies reduced autonomy over pregnancy desires and highlights how response measures may have potentially exacerbated the vulnerability of women and girls [38, 39]. Movement restrictions and other effects of COVID-19 that left men confined to the home may explain the rise in unintended pregnancies and the reported surge in demand for treatment of complications of abortion [7, 29]. Considering the legal, religious and moral landscape surrounding abortion in Burkina Faso [40], the lack of demand for the service is no surprise. Increased demand for treatment of complications of abortion indicates an increase in demand for clandestine abortions which, as evidence suggests are often unsafe [41]. This highlights the gendered effects of COVID-19, which reinforced the lack of women and girls’ autonomy over their SRH decisions.

Domestic movement restrictions and international travel bans disrupted supply chains leading to shortages in medicines and essential supplies [11, 29, 42]. In our study, supply chain related shortages presented a financial barrier to accessing contraceptives due to the need to purchase them from private pharmacies and could also explain the reported lack of personal protective equipment. Given the reported high stock-outs of commodities at baseline and endline, it is likely that COVID-19 worsened the chronic shortage of essential medicines and supplies, underscoring the need for general health system strengthening to address existing pre-pandemic challenges and contribute to better preparedness for future outbreaks. Travel restrictions and reduced public transportation also limited the ability to seek services [7, 38]. Furthermore, with men being confined to their homes most of the time due to movement restrictions, seeking family planning services was difficult for women, particularly covert contraceptive users. Reduced autonomy to seek family planning services has been linked to movement restrictions and stay-at-home orders [7, 29, 38]. This suggests the need for strengthening community based and mobile outreach services that would extend the reach of services beyond health facilities, mitigating the access barriers resulting from movement restrictions or disruptions in facility-based service delivery.

As predicted, global evidence suggests that reprioritisation of health services and reallocation of resources led to the reduction or suspension of non-essential services and staff shortages due to reassignment to COVID-19 treatment centres [15, 43, 44]. These, together with measures to reduce staff and patients’ exposure to infection and manage staff shortages [28, 44] disrupted delivery and utilisation of services. Our findings depart slightly, in that the disruptions were mostly partial and occurred during the period of high transmission and associated lockdown. This may reflect the relatively limited spread and shorter period of high transmission experienced in Burkina Faso. Although our findings show moderate to low domain scores for staffing and 43% of facilities reporting service disruption due to insufficient staff, this is likely a reflection of the pre-existing staff shortages, which may have worsened during the pandemic. Polis and colleagues reported that in low-and middle-income countries, albeit declines in contraceptive services, service disruptions occurred over a shorter duration, and few were severe [45]. Some of the available evidence suggests no substantial disruptions to family planning services [10, 11, 21, 22, 42], implying a lesser degree and shorter duration of service disruption than initially anticipated. The UNFPA attributed this to among others, resilient health systems and the limited spread of COVID-19 [46].

Despite the limited spread and lesser degree of service disruption, our findings highlight how the pandemic response may have increased barriers to family planning and abortion services, by exacerbating the negative effects of prevalent socio-cultural norms and unequal gender relations and further weakening the health system’s capacity to deliver services. This may potentially stagnate or reverse recent gains in Burkina Faso’s family planning indicators in the last decade: decline in fertility rate from 6.0 children per woman in 2010 to 5.2 in 2017, increased contraceptive use from 14% in 2010 to 29% in 2020 and decline in unmet need for family planning from 36.5% in 2015 to 20.2% in 2018 [41]. Interestingly, according to an analysis of Burkina Faso’s policy response to COVID-19, adaptations were made to counter the effects of containment measures and ensure the continuity of essential services, including the use of mobile clinics and community health workers to deliver home based care, task shifting and community level delivery of medicines/commodities [17]. Much of these adaptations are based on High Impact Practices (HIPs) for family planning [47], are aligned with lessons learned from the previous outbreaks [48, 49] and evidence from three African countries has demonstrated their effectiveness in mitigating COVID-19 related service disruptions [50]. Investigation into the implementation and effectiveness of the recommended adaptations may provide important lessons for public health emergency preparedness within the Burkina Faso context.

### Strengths and limitations

The mixed method approach used in this study enabled a comprehensive analysis of the barriers to availability, utilisation and readiness of SRH services during COVID-19. The inclusion of a qualitative approach places women’s rights and needs during health emergencies at the centre of the debate, underscoring the need for more responsive policies. Our study contributes to the growing body of evidence by strengthening the understanding of how COVID-19 and the pandemic response negatively affected delivery, demand and utilisation of family planning and abortion services.

The limitations are that our findings only represent women and girls from health facilities and do not include those in the community who did not seek services, which may have masked the effects of COVID-19 on demand and access to services. However, considering this was part of a wider study focusing on the health system availability, utilisation and readiness to deliver SRH services during COVID-19, our findings have provided important insights from the perspective of healthcare providers and clients.

## Conclusions

The COVID-19 pandemic necessitated the implementation of response measures, which while effective at mitigating the spread of infection, also resulted in disruptions in the demand, utilisation and delivery of family planning and abortion services. This may have exacerbated the existing barriers to services, and increased the vulnerability of women and girls, with potential long-term effects.

Government response to health emergencies should consider the effects of the containment measures on delivery and access to SRH services, particularly for women and girls. There is a need to strengthen the health system capacity to deliver family planning services during public health emergencies to ensure uninterrupted access in the context of movement restrictions and lockdowns.

## Supplementary Information

Additional file 1: Percentage of facilities with tracer items for family planning. The table lists the tracer items measured, the mean availability of tracer items, domain scores for family planning and percentage contraceptive commodity stockouts.

Additional file 2: Percentage of facilities with tracer items for Abortion. The table lists the tracer items measured, the mean availability of tracer items, domain scores for family planning and percentage abortion commodity stockouts.

## Data Availability

The data will be available upon request as per the WHO policies. Requests for access to data can be sent to alimoa@who.int.

## Abbreviations

CHU: University Teaching Hospital
CMA: Medical centre with surgical branch
COVID-19: Coronavirus Disease 2019
SRH: Sexual and Reproductive Health
UNFPA: United Nations Sexual and Reproductive Health Agency (United Nations Population Fund)
WHO: World Health Organization

## Declarations

### About This Supplement information

This article has been published as part of Reproductive Health, Volume 22 Supplement 03, 2025: The SRH Outbreak research response team within the Department of Sexual and Reproductive health. The full contents of the supplement are available at: https://link.springer.com/journal/12978/volumes-and-issues/22-3/supplement.
