# Supplementary material for: The dynamics of family planning and abortion services during COVID-19: perspectives from healthcare providers and clients in Burkina Faso
Source: Reprod Health. 2026 Jul 21;22(Suppl 3):278. doi: 10.1186/s12978-026-02340-x (PMC13417866; doi:10.1186/s12978-026-02340-x)
Supplement: Supplementary file 1 — Additional file 1: Percentage of facilities with tracer items for family planning. The table lists the tracer items measured, the mean availability of tracer items, domain scores for family planning and percentage contraceptive commodity stockouts. [file 12978_2026_2340_MOESM1_ESM.docx]

**Percentage of facilities with tracer items for family planning**

|  | **Tracer item** | **Baseline** | **Domain score** | **Endline** | **Domain score** |
| --- | --- | --- | --- | --- | --- |
| **1** | **Guidelines** | 100.0% | 100 | 87.5% | 87.5 |
| **2** | **Checklists and job aids** | 100.0% | 100 | 100.0% | 100 |
| **3** | **Referrals** | 0.0% | 0 | 0.0%^*^ | 0 |
|  | **Infrastructure** | | | | |
| 4 | Clear signs | 37.5% | 45.7 | 85.7%^*^ | 64.4 |
| 5 | Opening hours | 75% |  | 75% |  |
| 6 | Reception desk | 50.0% |  | 50.0% |  |
| 7 | Separate room for FPL | 75% |  | 87.5% |  |
| 8 | Separate w/room for adolescents | 0.0% |  | 0.0% |  |
| 9 | Counselling rooms curtained-off | 75% |  | 75.0% |  |
| 10 | Examination rooms curtained-off | 100.0% |  | 87.5% |  |
| 11 | Shelter for users | 75% |  | 87.5% |  |
| 12 | Written materials | 25.0% |  | 37.5% |  |
| 13 | Confidential records- clients contact | 100.0% |  | 100.0% |  |
| 14 | Confidential records- medical history | 100.0% |  | 87.5% |  |
| 15 | Confidential records- contraception history | 100.0% |  | 87.5% |  |
|  | **Commodities** | | | | |
| 16 | Facility stocks contraceptive commodities | 100.0% | 100 | 100.0% | 100 |
| 17 | Contraceptive commodities O/S: | **% facilities**  **reporting stock-outs** | **Average % of facilities reporting**  **stock-outs** | **% facilities**  **reporting stock-outs** | **Average % of facilities reporting**  **stock-outs** |
| 18 | Combined estrogen progesterone oral | 100.0% | 75.9 | 1000.0%^*^ | 89.8 |
| 19 | Progestin-only contraceptive pill | 87.4% |  | 85.7%^*^ |  |
| 20 | Combined estrogen progesterone injectable | 87.4% |  | 85.7%^*^ |  |
| 21 | Progestin-only injectable contraceptive | 100.0% |  | 100.0%^*^ |  |
| 22 | Male condom | 100.0% |  | 100.0%^*^ |  |
| 23 | Female condom | 100.0% |  | 100.0%^*^ |  |
| 24 | Emergency contraceptive pill | 50.0% |  | 85.7%^*^ |  |
| 25 | Cycle beads | 87.4% |  | 100.0%^*^ |  |
| 26 | Vaginal ring | 62.5% |  | 71.4%^*^ |  |
| 27 | Implant (sub dermal implant) | 87.4% |  | 85.7%^*^ |  |
| 28 | Copper IUD | 100.0% |  | 100.0%^*^ |  |
| 29 | Levonorgestrel IUD | 50.0% |  | 85.7%^*^ |  |
| 30 | Other natural family planning method | 25.0% |  | 85.7%^*^ |  |
| 31 | Diaphragm | 25.0% |  | 71.4%^*^ |  |
| 32 | Male sterilization available | 37.5% |  | 75.0%^*^ |  |
| 33 | Female sterilization available | 62.5% |  | 87.5%^*^ |  |
|  | **Human resources** | | | | |
| 34 | Training in FP | 62.5% | 69 | 87.5% | 50 |
| 35 | Training in adolescent SRH | 75% |  | 12.5% |  |

^*^Data available for 7 out of 8 facilities
