# Supplementary material for: The dynamics of family planning and abortion services during COVID-19: perspectives from healthcare providers and clients in Burkina Faso
Source: Reprod Health. 2026 Jul 21;22(Suppl 3):278. doi: 10.1186/s12978-026-02340-x (PMC13417866; doi:10.1186/s12978-026-02340-x)
Supplement: Supplementary file 2 — Additional file 2: Percentage of facilities with tracer items for Abortion. The table lists the tracer items measured, the mean availability of tracer items, domain scores for family planning and percentage abortion commodity stockouts. [file 12978_2026_2340_MOESM2_ESM.docx]

**Percentage of facilities with tracer items for abortion and postabortion care services**

| **Tracer Item** | | **Baseline** | **Domain score** | **Endline** | **Domain score** |
| --- | --- | --- | --- | --- | --- |
| National guidelines | | 100.0% | 100 | 85.7%^*^ | 86 |
| Checklist and job aids |  | 25.0% | 25 | 71.4%^*^ | 7 |
| Referrals | | 12.5% | 12.5 | 0.0%^*^ | 0 |
| **Infrastructure** | | | | | |
| Clear signs | | 14.3%^*^ | 55 | 42.9%^*^ | 63 |
| Opening hours | | 85.7%^*^ |  | 71.4%^*^ |  |
| Reception desk | | 62.5% |  | 42.9%^*^ |  |
| Separate room for ABO | | 25.0% |  | 57.1%^*^ |  |
| Separate w/room for adolescents | | 0.0% |  | 14.3%^*^ |  |
| Counselling rooms curtained-off | | 87.5% |  | 85.7%^*^ |  |
| Examination rooms curtained-off | | 87.5% |  | 85.7%^*^ |  |
| Shelter for users | | 85.7%^*^ |  | 42.9%^*^ |  |
| Written materials | | 0.0%^*^ |  | 14.3%^*^ |  |
| Confidential records- clients contact | | 100.0%^*^ |  | 100.0%^*^ |  |
| Confidential records- medical history | | 100.0%^*^ |  | 100.0%^*^ |  |
| Confidential records- contraception history | | 100.0%^*^ |  | 100.0%^*^ |  |
| **Commodities** | | | | | |
| Facility stocks abortion commodities | | 62.5% | 41 | 62.5% | 55 |
|  | | **% facilities**  **reporting stock-outs** | **Average % of facilities reporting**  **stock-outs** | **% facilities**  **reporting stock-outs** | **Average % of facilities reporting**  **stock-outs** |
| Abortion supplies O/S: | |  |  |  |  |
| 22-gauge spinal needles for paracervical block | | 62.5% |  | 75.0% |  |
| 21-gauge needles for drug administration | | 75.0% |  | 87.5% |  |
| Syringes 5 ml | | 87.5% |  | 87.5% |  |
| Syringes 10 ml | | 100.0% |  | 87.5% |  |
| Syringes 20 ml | | 87.5% |  | 87.5% |  |
| IV (intravenous) line | | 75.0% |  | 87.5% |  |
| Blood pressure equipment | | 87.5% |  | 87.5% |  |
| Stethoscope | | 87.5% |  | 87.5% |  |
| Speculum | | 100.0% |  | 87.5% |  |
| Tenaculum | | 75.0% |  | 75.0% |  |
| Tapered dilators up to 51 mm or equivalent circumference | | 62.5% |  | 87.5% |  |
| Electric vacuum aspirator (with 14 or 16 mm cannulae) | | 87.5% |  | 75.0% |  |
| MVA aspirator and cannulae up to 12 mm | | 87.5% |  | 75.0% |  |
| Uterine evacuation forceps | | 100.0% |  | 62.5% |  |
| Large, postpartum flexible curette | | 57.1%^*^ |  | 62.5% |  |
| Stainless steel bowl for preparing solution | | 100.0% |  | 87.5% |  |
| Instrument tray | | 75.0% |  | 87.5% |  |
| Clear glass dish for tissue inspection | | 50.0% |  | 62.5% |  |
| Oxygen and Ambu bag | | 62.5% |  | 75.0% |  |
| On-site access to an ultrasound machine (optional) | | 75.0% |  | 75.0% |  |
| Long needle-driver and suture | | 87.5% |  | 62.5% |  |
| Scissors | | 75.0% |  | 75.0% |  |
| Uterine packing | | 57.1%^*^ |  | 75.0% |  |
| Blood bank | | 42.9%^*^ |  | 62.5% |  |
| Medicines O/S: | | **% facilities**  **reporting stock-outs** | **Average % of facilities reporting**  **stock-outs** | **% facilities**  **reporting stock-outs** | **Average % of facilities reporting**  **stock-outs** |
| Misoprostol | | 50.0% |  | 50.0% |  |
| Osmotic dilators | | 62.5% |  | 50.0% |  |
| Mifepristone | | 37.5% |  | 37.5% |  |
| Analgesics | | 25.0% |  | 12.5% |  |
| Anxiolytics | | 0.0% |  | 0.0% |  |
| Antibiotics | | 25.0% |  | 25.0% |  |
| Fluids (saline, sodium lactate, glucose) | | 0.0% |  | 25.0% |  |
| Lidocaine for paracervical block | | 25.0% |  | 62.5% |  |
| Appropriate antagonists to medications used for pain | | 0.0% |  | 62.5% |  |
| Uterotonics (oxytocin, misoprostol or ergometrine) | | 12.5% |  | 62.5% |  |
| Antiseptic solution (non-alcohol based) to prepare the cervix | | 0.0% |  | 62.5% |  |
| Sterilization or high-level disinfection solutions and materials | | 0.0% |  | 12.5% |  |
| Sundries lacking: | | **% facilities**  **lacking** | **Average % of facilities lacking** | **% facilities**  **lacking** | **Average % of facilities lacking** |
| Clean examination gloves | | 12.5% |  | 12.5% |  |
| Gown | | 12.5% |  | 100.0% |  |
| Face protection | | 62.5% |  | 3.75% |  |
| Clean water | | 25.0% |  | 25.0% |  |
| Detergent or soap | | 12.5% |  | 25.0% |  |
| Instrument soaking solution | | 25.0% |  | 12.5% |  |
| Gauze sponges or cotton balls | | 37.5% |  | 25.0% |  |
| Sanitary napkins or cotton wool | | 62.5% |  | 25.0% |  |
| Strainer (metal, glass or gauze) | | 42.9%^*^ |  | 12.5% |  |
| Adequate toilet facilities | | 50.0% |  | 12.5% |  |
| Printed information for clients on post-procedure self-care | | 62.5% |  | 3.75% |  |
| Clear referral mechanisms to higher-level facility, when needed | | 25.0% |  | 3.75% |  |
| Private area with chairs separate from antenatal or labour care, for women who wait in clinic for expulsion | | 62.5% |  | 3.75% |  |
| **Human resources** | | | | | |
| Training in safe abortion | | 28.6%^*^ | 22 | 12.5% | 13 |
| Training in adolescent SRH | | 14.3%^*^ |  | 14.3%^*^ |  |

*Data available for less than 8 facilities
